# The Impacts of the COVID-19 Pandemic on Family Caregivers' Care Work, Anxiety, and Loneliness

**DOI:** 10.1093/geroni/igab046.2108

**Published:** 2021-12-17

**Authors:** Sharon Anderson, Jasneet Parmar

**Affiliations:** 1 Faculty of Medicine & Dentistry University of Alberta, Edmonton, Alberta, Canada; 2 Department of Family Medicine, University of Alberta, Edmonton, Alberta, Canada

## Abstract

Our study examined the effects of COVID-19 pandemic and public health measures on family caregivers (FCGs) of frail older adults; specifically, their care work, anxiety, and loneliness all of which are associated with decreased wellbeing. Approximately 604 FCGs completed the survey and findings evidenced COVID-19 creating two solitudes. First, 73% of FCGs for individuals living with them were providing significantly more care during COVID-19. Second, those caring for residents in congregate settings were unable to care. Both situations, community-dwelling and congregate care, increased FCG distress and decreased wellbeing. Anxiety significantly increased from 36% pre COVID-19 to 54% during COVID-19. Loneliness increased from 46% to 85%. FCGs report their mental (58%) and physical (48%) health deteriorated. The detrimental impact of the pandemic and public health measures on FCGs caring at home and in congregate care, and their related needs, need immediate attention from both the health and social systems of care.

